# Ameliorated effects of a lipopeptide surfactin on insulin resistance in vitro and in vivo

**DOI:** 10.1002/fsn3.2852

**Published:** 2022-03-29

**Authors:** Xiaoyu Chen, Hongyuan Zhao, Fanqiang Meng, Libang Zhou, Xinyi Pang, Zhaoxin Lu, Yingjian Lu

**Affiliations:** ^1^ College of Food Science and Technology Nanjing Agricultural University Nanjing Jiangsu Province China; ^2^ College of Food Science and Engineering Nanjing University of Finance and Economics Nanjing Jiangsu Province China

**Keywords:** GLUT4, inflammation, insulin resistance, oxidative stress, PI3K/Akt pathway, surfactin

## Abstract

Surfactin, produced by *Bacillus amyloliquefaciens* fmb50, was used to treat insulin‐resistant (IR) hepatocyte. It was found that surfactin increased glucose consumption in insulin‐resistant HepG2 (IR‐HepG2) cells and ameliorated IR by increasing glucose transporter 4 (GLUT4) protein expression and AMP‐activated protein kinase (AMPK) mRNA expression, promoting GLUT4 translocation and activating phosphatidylinositol 3‐kinase (PI3K)/protein kinase B (Akt) in IR‐HepG2 cells. Meanwhile, surfactin downregulated protein expression of phosphoenolpyruvate carboxy kinase (PEPCK) and glucose‐6‐phosphatase (G6Pase), further inhibiting hepatic gluconeogenesis. In addition, surfactin played important roles in eliminating reactive oxygen species (ROS), improving mitochondrial dysfunction, and inhibiting proinflammatory mediators. We observed that surfactin promoted glucose consumption, meanwhile increased translocation and protein expression of GLUT4 in Caco‐2 cells. These results confirmed the conclusion in hepatic cells. Furthermore, surfactin supplement decreased body weight, food intake, and fasting blood glucose of type 2 diabetes mellitus (T2DM) mice induced by streptozotocin (STZ)/high‐fat diet (HFD). Our data indicated that surfactin ameliorated insulin resistance and lowered blood glucose in intro and in vivo.

## Graphical abstract

Surfactin ameliorated IR by inhibiting gluconeogenesis, oxidative stress, mitochondrial damage, and proinflammatory factors in HepG2 cells, meanwhile promoted glucose consumption by increasing protein expression and translocation of GLUT4 in Caco‐2 cells. In addition, surfactin also ameliorated T2DM through lowering body weight, food intake, and fasting blood glucose mice induced by STZ/HFD.

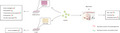

## 1 INTRODUCTION

At present, much attention is drawn by food‐derived bioactive substances for alleviating insulin resistance (IR) owing to their nontoxic, non–side effects. It is reported that food‐derived peptides possess beneficial effects against IR and diabetes (Lv et al., 2019; Wang et al., 2020a, 2020b). The walnut‐derived peptide LPLLR alleviated IR by activating IRS‐1/PI3K/Akt and AMPK signaling pathways (Wang et al., 2020a). In addition, the postbiotics, known as bacterial or biogenic metabolites, such as microbe‐derived peptides, also improved obesity‐induced metabolic disorders, including hyperglycemia and hypertension (Zouari et al., 2016, 2017). The peptide from *Bacillus subtilis* decreased blood glucose and α‐amylase activity and protected β‐cells from damage in diabetic rats (Zouari et al., 2015). A lipopeptide surfactin showed a potent antidiabetic effect on type 1 diabetes mellitus (T1DM) in nonobese diabetic (NOD) mice (Gao et al., 2014). Recently, it was found that surfactin could reduce the levels of proinflammatory factors, suppress oxidative stress, and improve hepatic function by downregulating protein expression of interleukin‐1β (IL‐1β), interleukin‐6 (IL‐6), and tumor necrosis factor‐α (TNF‐α) and upregulating protein expression of interleukine‐10 (IL‐10) (Wang et al., 2021). Although a lipopeptide surfactin has a wide range of biological activity, such as antibacterial, antiviral, anti‐inflammatory, and thrombolytic activity (Wang et al., 2021), few studies focused on its regulation in glucose metabolism.

Diabetes mellitus (DM) is a major cause of morbidity and mortality in the modern era and also decreases both quality of life and life expectancy (Riaz et al., 2020). Increased blood glucose level is a typical symptom for patients with diabetes. Metabolic glucose absorption in target cells is dependent on the insulin and glucagon levels, which are mainly secreted from the pancreatic islet cells. Type 1 diabetes mellitus (T1DM) is characterized by an insufficient insulin level, while type 2 diabetes mellitus (T2DM) is accompanied by insulin resistance (IR) (Riaz et al., 2020) and dysregulation of glucose utilization. It is well known that IR is often accompanied with ROS overproduction, which leads to oxidative stress in the liver. In normal state, high blood glucose level induces β cells to synthesize insulin. If persisting, this overwhelms the endoplasmic reticulum capacity and leads to the accumulation of misfolded protein (Riaz et al., 2020). This disturbed endoplasmic reticulum environment induces islets β‐cell impairment and insulin resistance or production insufficient, leading to the efficiency of insulin‐mediated glucose uptake and utilization reduction and glucose regulation disorders in the liver (Chen et al., 2019; Jiang et al., 2014). In addition, IRE1α plays a major role in insulin biosynthesis and keeping the oxidative balance in islets β cell (Hassler et al., 2015). Severe high glucose could promote dephosphorylation of IRE1α, resulting in the attenuation of IRE1α activity and insulin resistance or reduced insulin production (Qiu et al., 2010). Therefore, amelioration of hepatic IR is regarded as an important strategy in the prevention and treatment of T2DM.

It was reported that mitochondrial dysfunction can also stimulate excessive ROS generation (Tang et al., 2020). Furthermore, PI3K, Akt, AMPK, and GLUT4 are closely involved in the regulation of glucose uptake and metabolism (Lv et al., 2019; Wang, He, et al., 2020). In particular, AMPK, a phylogenetically conserved intracellular energy sensor, can regulate insulin sensitivity in the liver (Ren et al., 2018), and it is a potential target for ameliorating IR. GLUT4 translocation from cytoplasm to membrane surface is basal and essential for glucose transport and uptake. Food‐derived substances also exhibited the effects of alleviation IR via above signaling pathways. *Dendrobium officinale* polysaccharide could increase protein expression of PI3K, Akt, and GLUT4 to improve glucose metabolism disorder (Wang et al., 2018). Zhenjiang aromatic vinegar extract exhibited positive effects on elimination ROS through phosphorylating c‐Jun N‐terminal kinase (JNK) in IR‐HepG2 cells (Xia et al., 2021). It is reported that glucose metabolism disorder can be improved by activating the IRS‐1/PI3K/Akt and AMPK signaling pathways in hepatocytes (Ren et al., 2018; Wang et al., 2020b). Two gluconeogenic enzymes, including PEPCK and G6Pase, were suppressed by upregulating p‐Akt (Ren et al., 2018). It is demonstrated that glucose uptake in insulin‐sensitive tissues can be promoted by activating insulin‐mediated signaling pathways or directly upregulating GLUT4 protein expression without enhancing insulin signaling (Choi et al., 2011).

In this study, the effects of a lipopeptide surfactin on high insulin‐induced IR in HepG2 cells were explored. Moreover, the effects of surfactin on insulin signaling pathways, gluconeogenic enzymes, inflammatory factors, and ROS level were investigated in HepG2 cells. Subsequently, the effects of surfactin on glucose consumption in Caco‐2 were further confirmed. Furthermore, the effects of surfactin on T2DM mice induced by STZ/HFD were investigated.

## 2 MATERIALS AND METHODS

### 2.1 Materials and reagents

*Bacillus amyloliquefaciens* fmb50 was obtained from the laboratory of Enzyme Engineering, College of Food Science and Technology in Nanjing Agricultural University (Yao et al., 2015; Zhang et al., 2014). Dulbecco's modified Eagle's medium (DMEM) and fetal bovine serum (FBS) were purchased from Gibco Co., Ltd. The insulin was purchased from Shanghai Aladdin Biochemical Technology Co., Ltd. The enzyme‐linked immunosorbent assay (ELISA) kits for AMPK, glycerol kinase (GK), GLUT4, PEPCK, G6Pase, and glycogen were purchased from Feiya Biotechnology Institute. The commercial kit for ROS was purchased from Keygen Biotech Institute, and mitochondrial membrane potential kit was purchased from Nanjing Sciben Biotech Co., Ltd. Membrane and cytoplasmic proteins extraction kit was purchased from Nanjing Jiancheng Bioengineering Institute. Anti‐GLUT4 primary antibody was purchased from Boster Biological Technology Co., Ltd. 4′,6‐diamidino‐2‐phenylindole (DAPI) was purchased from Beyotime Biotechnology Co., Ltd. Streptozotocin (STZ) was purchased from Shanghai Aladdin Biochemical Technology Co., Ltd. The primary antibodies against PI3K, Akt, AMPK, phosphoinositide‐PI3K (p‐PI3K), phosphoinositide‐Akt (p‐Akt), and glyceraldehyde 3‐phosphate dehydrogenase (GAPDH) were purchased from Affinity Biosciences. The primary antibodies against phosphoinositide‐AMPK (p‐AMPK) were purchased from Beyotime Institute of Biotechnology. All other analytical reagents were purchased from Sinopharm Chemical Reagent Co., Ltd.

### 2.2 Fermentation and production of surfactin from *Bacillus amyloliquefaciens* fmb50

Surfactin samples were obtained from fermentation broths followed by flocculating (0.5 g/L chitosan and 0.3 g/L sodium alginate, pH = 5.0), dissolving (absolute ethanol), and freeze drying. The surfactin sample was identified using high‐performance liquid chromatography (HPLC) (U‐3000, Dionex) equipped with an Agilent C18 column (4.5 mm × 250 mm, Agilent) and a UV detector (Tang et al., 2020). The collected surfactin dry powder was stored at 4°C for the following experiments.

### 2.3 Cell culture and treatments

HepG2 cells were obtained from the cell bank of Chinese Academy of Science and cultured in high‐glucose (4.5 g/L) DMEM supplemented with 10% FBS and 1% penicillin–streptomycin solution under 5% CO_2_ at 37°C. After activation, all cells used in experiments were within 20 generations. An insulin‐induced IR‐HepG2 cells model was established using high‐glucose medium according to the previous method (Wang et al., 2019a) with slight modification. Briefly, when HepG2 cells reached over 80% confluence, the cells were cultured in a medium with normal DMEM (10% FBS and 1% penicillin–streptomycin mixed solution) as the control group, cells were treated with 15 µg/ml insulin for 48 h as the model group (IR‐HepG2 cells), and cells were treated with insulin and different concentrations of surfactin (6.25, 12.5, 25, 50, and100 µg/ml) as the surfactin group.

Caco‐2 cells were obtained from the Cell Bank of Chinese Academy of Science (Shanghai, China) and cultured in DMEM supplemented with 20% FBS and 1% penicillin–streptomycin solution under 5% CO_2_ at 37°C. Cells were cultured with DMEM (20% FBS and 1% penicillin–streptomycin mixed solution) for 48 h as the control group, and cells were treated with different concentrations of surfactin (6.25, 12.5, 25, 50, and 100 µg/ml) as the surfactin group.

### 2.4 Cell viability assay

Cell viability was assessed according to a previous literature (Wang & Dong, 2019b). In brief, HepG2 and Caco‐2 cells were seeded in 96‐well plates at a density of 3 × 10^3^ cells/well and incubated for 48 h. Cells were treated with 15 µg/ml of insulin and various concentrations of surfactin (6.25, 12.5, 25, 50, and 100 µg/ml). Then, the culture medium was removed and 100 µl of 1 mg/ml MTT was added into each well and incubated for an additional 4 h at 37°C. Subsequently, 50 µl of dimethyl sulfoxide (DMSO) was added into each well after removing the MTT solution, and the absorbance at 490 nm was detected by a microplate reader. The absorbance of the control group was regarded as 100%. The cell viability was calculated using the following equation:

$$\text{Cell viability}\;\left(\%\right)\;=\;\frac{{\text{OD}}_{\text{treat}}}{{\text{OD}}_{\text{control}}}\times 100\%$$

### 2.5 Glucose consumption assay

HepG2 cells were seeded in 96‐well plates and treated with insulin and different concentrations of surfactin. After discarding medium, 200 µl FBS‐free medium was added and cells were incubated for 12 h. Caco‐2 cells were seeded in 96‐well plates and treated without or with different concentrations of surfactin. The glucose level in collected supernatant was determined by a glucose assay kit (Beijing Applygen Gene Technology Co., Ltd). The absorbance values were measured at 550 nm. MTT analysis was used to adjust the glucose consumption. The glucose consumption was calculated by the following equation:

Glucose consumption = initial glucose concentration in the medium (no cells, only medium) – terminal glucose concentration in each group (control, model, and treatment groups). The relative levels of glucose consumption in HepG2 cells were normalized by cell viability as glucose consumption/cell viability.

### 2.6 Measurement of intracellular ROS generation

HepG2 cells were seeded in six‐well plates. Intracellular ROS levels were determined by a fluorescent probe 2′,7′‐dichlorofluorescein‐diacetate (DCFH‐DA) (Keygen biotech Institute). Firstly, 10 µM DCFH‐DA was added to each well and cells were incubated at 37°C for 30 min in the dark. Then, the cells were washed three times with phosphate‐buffered saline (PBS), and fluorescence intensity was measured by flow cytometry within 30 min.

### 2.7 Determination of mitochondrial membrane potential

HepG2 and Caco‐2 cells were seeded in six‐well plates. Detection of mitochondrial membrane potential was carried out using fluorescent probe JC‐1, according to manufacturer's instructions (Nanjing Sciben Biotech Co., Ltd). Cells were incubated with FBS‐free medium containing JC‐1 probe for 20 min, and then cells were washed two times using JC‐1 staining buffer to discard excess JC‐1 probe. Fluorescence images in different groups were captured by Fluorescence Imager (Nikon Eclipse 80i, Nikon Co.). The image J was applied to analyze the fluorescence intensity of fluorescence images. In addition, when the mitochondrial membrane potential is high, JC‐1 is driven into mitochondrial matrix to form JC‐1 aggregates, and thus, the intensity of red fluorescence is dramatically increased. By contrast, when the mitochondrial membrane potential is low, JC exists as a monomer and it exhibits green fluorescence. The mitochondrial membrane potential was calculated using the following equation:

Mitochondrial membrane potential = the fluorescence intensity of JC‐1 aggregate/the fluorescence intensity of JC‐1 monomer (Du et al., 2010).

### 2.8 RT‐PCR analysis

HepG2 and Caco‐2 cells were cultured in six‐well plates. After culturing, total RNA in cells was extracted using commercial kit (TransGen Biotech. Co., Ltd) according to manufacturer's instruction. The concentrations of the extracted RNA were measured using Nanodrop 2000 (Thermo Ficher Scientific Inc.). The extracted RNA (200–800 ng/µl) was reverse transcribed into cDNA using a HiScript II Q RT SuperMix kit (Vazyme Biotech. Co., Ltd.). The primer sequences of target genes were listed in Table 1. RT‐PCR analysis was performed using HieffTM SYBR Green Master Mix (Yeasen Biotech Co., Ltd.) and a RT‐PCR detection system (Applied Biosystem). About 100 ng cDNA was used to measure the mRNA levels of target genes. The detailed program was as follows: 95°C for 120 s; then, 40 cycles of 10 s at 95°C, 30 s at 60°C, and 30 s at 72°C for extension, and the melting curve was applied to check the specificity of the primers. The data were normalized to mRNA expression of glyceraldehyde‐3‐phosphate dehydrogenase (GAPDH), which was used as an endogenous control. The relative expression level of genes was calculated using the 2^−ΔΔCt^ method. In addition, RT‐PCR analysis was carried out using 6.25–25 µg/ml of surfactin.

**TABLE 1:** The primers for quantitative of RT‐PCR

Genes	Forward primer (5′→3′)	Reserve primer (5′→3′)
GLUT4	TGGAAGGAAAAGGGCCATGCTG	CAATGAGGAATCGTCCAAGGATG
PI3K	TTAAACGCGAAGGCAACGA	CAGTCTCCTCCTGCTGTCGAT
Akt	CAAGCCCAAGCACCGT	GAATCACCTTCCCAAAGGTG
AMPK	GCCTGCCACAGACACCACTT	GCCACAGGGTGACACAGGAG
HNF4α	CACTACGGTGCCTCGAGCTG	CGGTCCCGCTCATTCTGGAC
IL−1β	CACAGCAGCACATCAACAAG	GTGCTCATGTCCTCATCCTG
IL−6	AGCTGCAGGCACAGAACCAG	TCTGTGCCCAGTGGACAGGT
TNF‐α	CCTCTCTCTAATCAGCCCTCTG	GAGGACCTGGGAGTAGATGAG
Regulator of GLUT4	TCTCGGGGCCTCTGGATCTG	TCCACGTTGCCCTGTTGCAT
GAPDH	GCACCGTCAAGGCTGAGAAC	TGGTGAAGACGCCAGTGGA

### 2.9 Measurement of parameters involved to glucose metabolism

The production of glycogen, and the protein contents of AMPK, GK, GLUT4, PEPCK, and G6Pase were determined using ELISA kits (Feiya Biotechnology Institute), according to manufacturers’ instructions. In addition, the levels of glycogen and GK were shown in Figure S1(a,b).

### 2.10 Extraction and measurement of GLUT4 in the membrane and cytoplasm

Extraction of protein in membrane and cytoplasm in HepG2 and Caco‐2 cells was carried out using commercial kit (Nanjing Jiancheng Bioengineering Institute), according to the manufacturer's instruction. Briefly, the total protein in the membrane and cytoplasm was extracted separately using differential centrifugation, according to the manufacturer's instruction. Subsequently, protein GLUT4 in the membrane and cytoplasm was determined using ELISA kit (Feiya Biotechnology Institute).

### 2.11 Immunofluorescence staining for GLUT4

In HepG2 and Caco‐2 cells, GLUT4 was observed by immunofluorescence staining. Briefly, the cells were treated without or with surfactin, and washed with PBS buffer for three times and fixed using 4% (w/v) paraformaldehyde for 8 min. Cells were blocked using 1% goat serum albumin (GSA) at room temperature for 1 h (Wang et al., 2021). Subsequently, cells were treated with anti‐GLUT4 primary antibody overnight at 4°C. Cells were incubated with goat anti‐rabbit IgG/FITC secondary antibody at room temperature for 1 h, and then treated with DAPI (2 µg/ml) for 8 min in the dark. Finally, the cells were washed with PBS buffer for three times. In addition, a control for the immunofluorescence GLUT4 was performed only through replacing anti‐GLUT4 primary antibody by PBS buffer. Fluorescence imaging was performed using a Fluorescence Imager (Nikon Eclipse 80i, Nikon Co.). The image J was applied to analyze the fluorescence intensity of fluorescence images.

### 2.12 Western blot analysis

Total proteins in HepG2 cells were extracted using radioimmunoprecipitation assay (RIPA) buffer added with 1 mM phenylmethylsulfonyl fluoride (PMSF) (Beyotime Institute of Biotechnology). The supernatant of whole‐cell lysates was collected by centrifugation at 12,000 ***g*** for 15 min at 4°C, and used for western blot. Proteins were separated using 8% and 12% sodium dodecyl sulfate–polyacrylamide gels (SDS‐PAGE), which then was transferred to nitrocellulose (NC) membrane (GE Healthcare Life Science). After blocking in Tris‐buffered saline and 0.1% Tween 20 (TBST) supplemented with 5% skim dry milk for 2 h, the membranes were overnight incubated with diluent primary rabbit antibodies at 4°C. After washing with TBST solution for three times, the membranes were incubated with HRP‐linked secondary antibody anti‐rabbit IgG (Beyotime Institute of Biotechnology) for 2 h. Finally, the bands were scanned using ECL plus solution (Affinity Biosciences). Target band density was quantified using Image J software (National Institutes of Health). GAPDH was the internal standard.

### 2.13 Animal experimental design

Totally, 50 health 4‐week male Kunming mice were provided by Beijing Vital River Laboratory Animal Technology Co., Ltd. All the animals were kept on constant conditions (temperature 25°C; 12‐h light–dark cycle) in aseptic environments, and fed with basic diet and water randomly to acclimatize for 7 days. Subsequently, mice were divided into five groups randomly, each containing 10 mice. The control group was fed with basic diet, and the T2DM group and surfactin treatment group were fed with 60% high‐fat diet (HFD) (Xietong CO.) for 12 weeks. In addition, surfactin treatment groups were administrated with 300 μl of surfactin (40 mg/kg· body weight as the LSF group, 80 mg/kg·body weight as the MSF group, and 160 mg/kg· body weight as the HSF group) after acclimatizing for 7 days. The control and T2DM group were administrated with 300 μl of 0.9% physiological saline.

After feeding for 4 weeks, mice were fasted for 12 h, and then fasting blood glucose (FBG) was measured before induction with STZ. The mice in the T2DM and surfactin treatment group received a double intraperitoneal injection of 30 mg/kg STZ at intervals of 1 week. The control group was injected with equivalent volume of citrate buffer. After STZ injection for 3 days, FBG of all mice was measured by the tail tip. The mice with FBG ≥ 7.8 mmol/L were marked as T2DM animals (Lv et al., 2010).

### 2.14 Measurement of body weight, food intake, and fasting blood glucose

Body weight and food intake were monitored every 7 days. The fasting blood glucose (FBG) was determined from 12‐h fasting mice for every 2 weeks during experiments.

### 2.15 Statistical analysis

The data showed as mean ± standard derivation (*SD*). Variances among the groups were analyzed by one‐way analysis of variance (anova) in SPSS statistics followed by Duncan test. *p* < .05 indicated significant differences.

## 3 RESULTS

### 3.1 Effects of surfactin on cell viability of IR‐HepG2 cells

**FIGURE 1:**
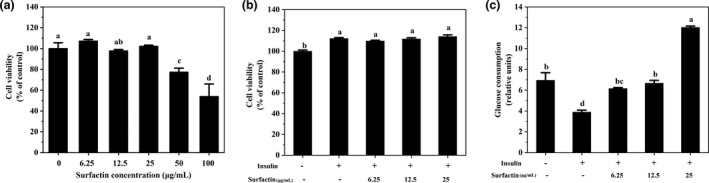
Effect of surfactin on cell viability and glucose consumption in IR‐HepG2. (a) Cell viability with different concentrations (0–100 μg/ml) of surfactin for 48 h. (b) Cell viability with insulin (15 μg/ml) and different concentrations (6.25–25 μg/ml) of surfactin for 48 h. (c) Glucose consumption with insulin (15 μg/ml) and different concentrations (6.25–25 μg/ml) of surfactin for 48 h. All data are expressed as mean ± *SD* (*n* = 6) for each group. Different lowercase alphabet letters were significantly different at level of *p* < .05

To evaluate cytotoxic effects of surfactin on hepatic cells, cell viability was examined by MTT assay. The results exhibited that 6.25–25 µg/ml of surfactin had no negative effect on cell viability in HepG2 and IR‐HepG2 cells as compared with that in the control group (Figure 1a,b), illustrating that surfactin had no cytotoxic effect on HepG2 cells at selected concentrations.

### 3.2 Effects of surfactin on the glucose consumption in IR‐HepG2 cells

To investigate the potential roles of surfactin in IR‐HepG2 cells, the glucose consumption was detected in HepG2 cells. As shown in Figure 1(c), the glucose consumption in IR‐HepG2 cells were significantly decreased as compared with that in the control group, indicating that insulin resistance could inhibit glucose consumption. However, it was found that surfactin treatment significantly increased glucose consumption in IR‐HepG2 cells, especially at high dose (25 µg/ml) (Figure 1c). These results suggested that surfactin was able to enhance glucose consumption and reduce insulin resistance in IR‐HepG2 cells. In addition, 6.25 µg/ml of surfactin as surfactin group was used for the following study in HepG2 cells.

### 3.3 Effects of surfactin on GLUT4 in IR‐HepG2 cells

**FIGURE 2:**
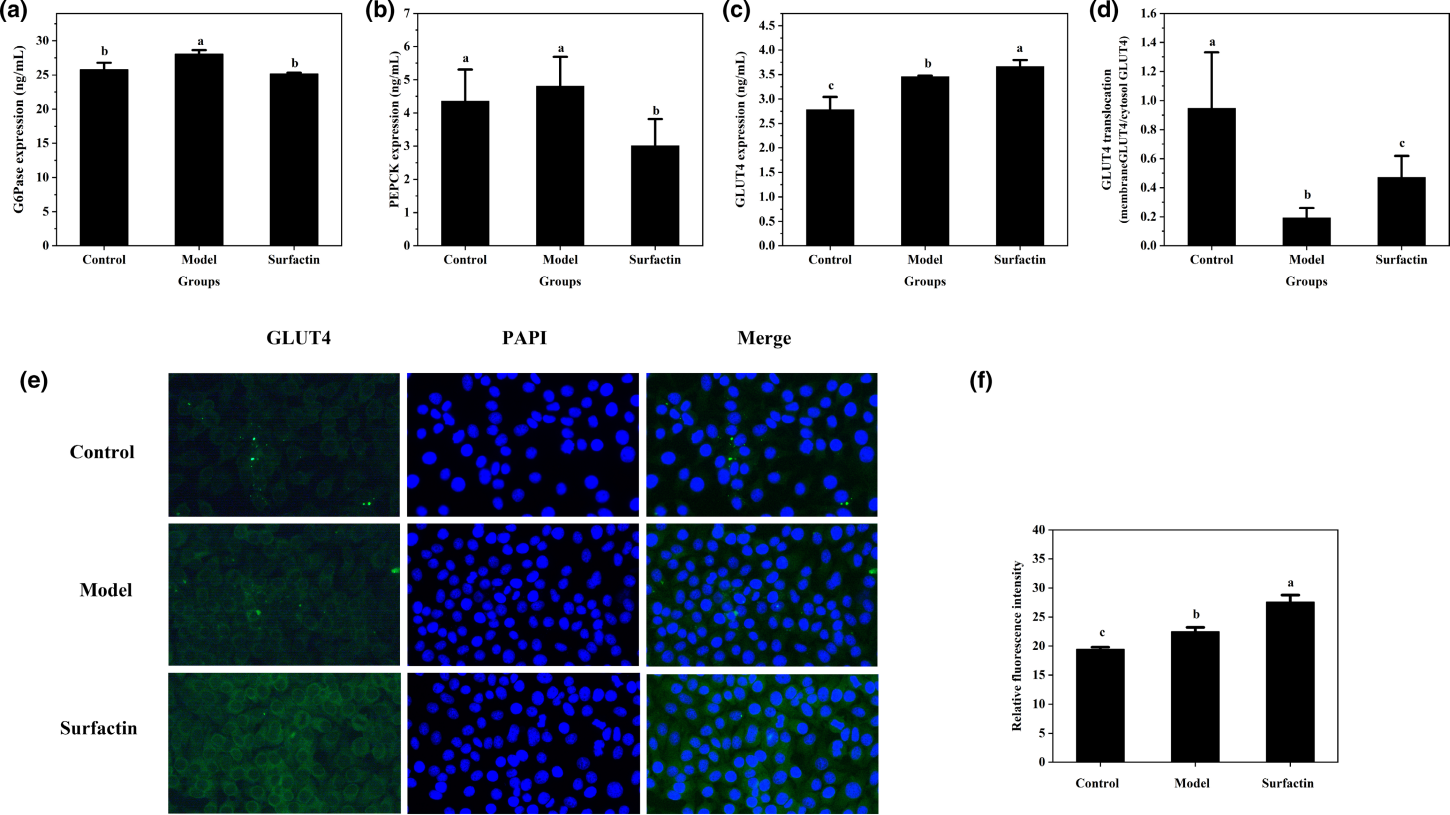
Effects of surfactin on gluconeogenesis‐related enzymes and GLUT4 translocation in IR‐HepG2 cells. (a) The protein expression of G6pase. (b) The protein expression of PEPCK. (c) The protein expression of GLUT4. (d) The GLUT4 translocation. (e) Representative images of immunofluorescence staining of GLUT4. (f) The corresponding relative fluorescence intensity of GLUT4. All data are presented as mean ± *SD* (*n* = 3) for each group. Different lowercase alphabet letters over bars indicate statistically significant differences between two groups (*p* < .05)

The protein expression of GLUT4 was detected to further explore whether surfactin improved glucose consumption by promoting protein expression of GLUT4 to the membrane. Figure 2(c) showed that the protein expression of GLUT4 was significantly increased in IR‐HepG2 cells as compared with that in control group (*p* < .05), and surfactin significantly promoted protein expression of GLUT4 when compared with that in IR‐HepG2. The immunofluorescence staining analysis also showed that surfactin increased protein expression of GLUT4 (Figure 2e,f).

In addition, the translocation of GLUT4 was also determined by measuring the protein expression of GLUT4 in both the membrane and cytoplasm. As shown in Figure 2(d), the membrane GLUT4/cytosol GLUT4 ratio in IR‐HepG2 group was dropped by 79.6% as compared with that in the control group (*p* < .05), leading to a reduction in cellular glucose consumption (Figure 1e). Surfactin treatment (6.25 µg/ml) elevated the GLUT4 translocation by 144.1% as compared with that in IR‐HepG2 group (*p* < .05). Several similar phenomena were also observed by Jang et al. (2010), and Wang et al. (2020a); GLUT4 translocation and glucose consumption in insulin‐resistant HepG2 cells were increased by novel peptides from black soybean and *walnut*.

### 3.4 Effects of surfactin on gluconeogenic key enzymes in IR‐HepG2 cells

It is well known that G6Pase and PEPCK are key rate‐limiting enzymes in hepatic gluconeogenesis process (Wang & Dong, 2019b). To investigate the effect of surfactin on gluconeogenesis in IR‐HepG2 cells, the protein expression of G6Pase and PEPCK was detected. The results showed that high insulin administration dramatically upregulated protein expression of G6Pase (*p* < .05), while had no significant effect on protein expression of PEPCK in IR‐HepG2 cells (*p* > .05). However, the protein expression of G6Pase and PEPCK was markedly downregulated after surfactin treatment as compared with those in IR‐HepG2 cells (*p* < .05) (Figure 2a,b). Furthermore, these results indicated that surfactin can inhibit hepatic gluconeogenesis through suppressing protein expression of G6Pase and PEPCK.

### 3.5 Effects of surfactin on key genes and protein involved in glucose metabolism in IR‐HepG2 cells

**FIGURE 3:**
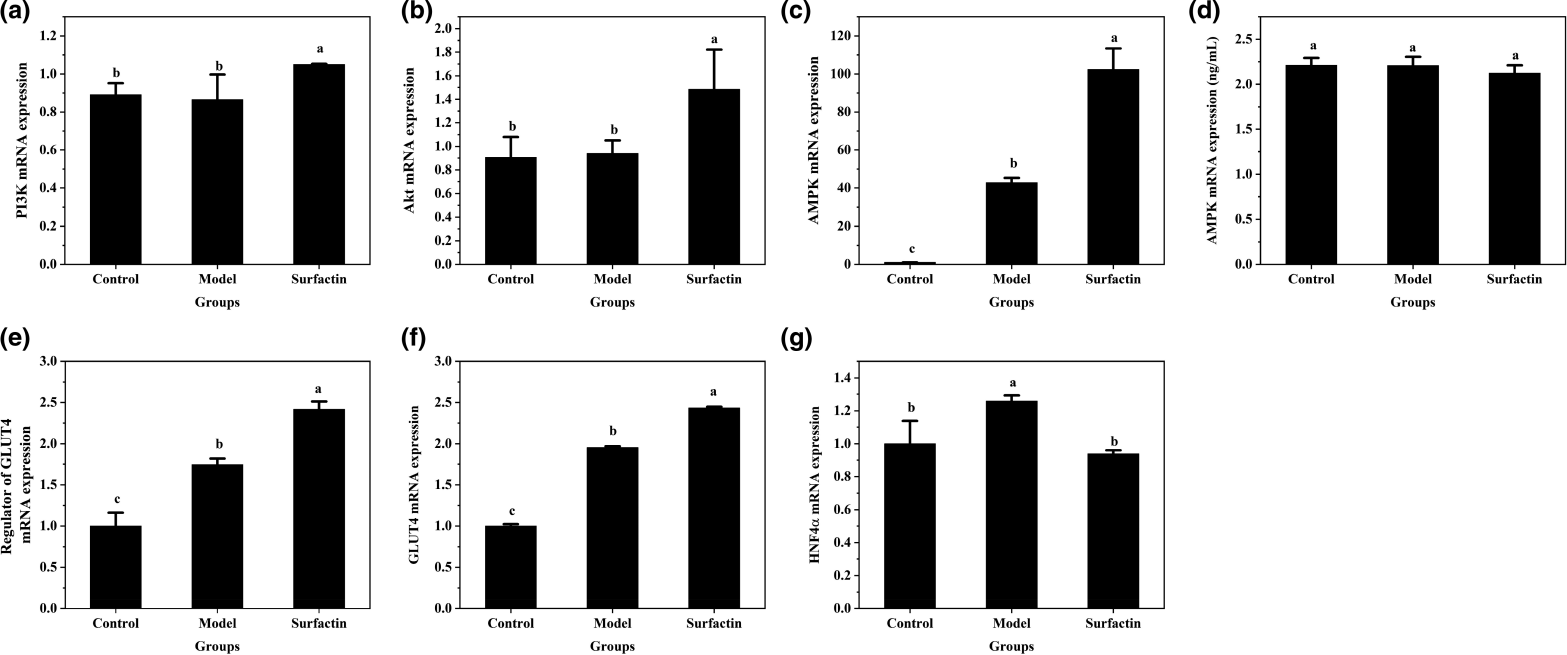
Effects of surfactin on mRNA expression levels of key genes in insulin signaling pathway and gluconeogenesis in IR‐HepG2 cells. (a) The mRNA expression of PI3K. (b) The mRNA expression of Akt. (c) The mRNA expression of AMPK. (d) The protein expression of AMPK. (e) The mRNA expression of regulator of GLUT4. (f) The mRNA expression of GLUT4. (g) The mRNA expression of HNF4α. All data are presented as mean ± *SD*, (*n* ≥ 3) for each group. Different lowercase alphabet letters over bars indicate statistically significant differences between two groups (*p* < .05)

**FIGURE 4:**
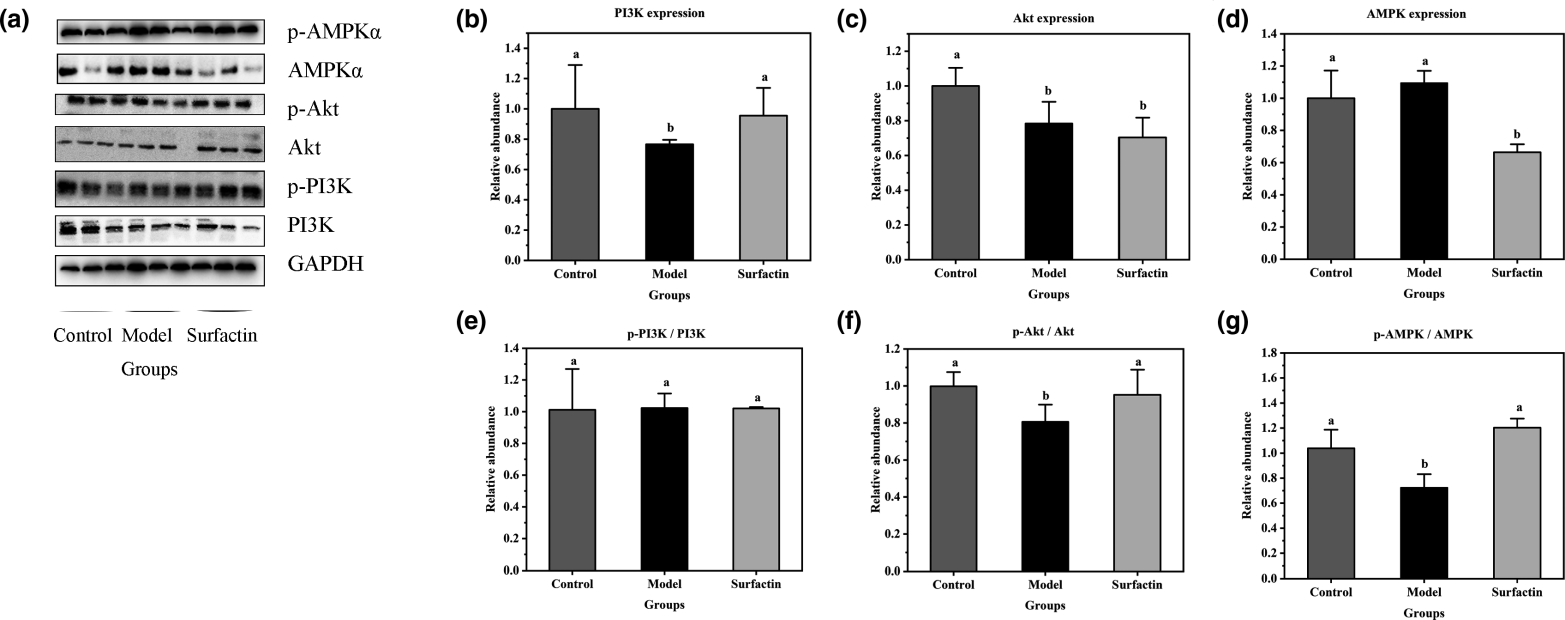
Effects of surfactin on key protein in insulin signaling pathway in IR‐HepG2 cells. (a) The western blot analysis. (b) The protein expression of PI3K. (c) The protein expression of Akt. (d) The protein expression of AMPK. (e) The ratio of p‐PI3K to PI3K. (f) The ratio of p‐Akt to Akt. (g) The ratio of p‐AMPK to AMPK. All data are presented as mean ± *SD* (*n* ≥ 3) for each group. Different lowercase alphabet letters over bars indicate statistically significant differences between two groups (*p* < .05)

It is reported that GLUT4 translocation from cytoplasm to membrane surface is mainly regulated by the IRS‐1/PI3K/Akt signaling pathways (Guo et al., 2019), and the phosphorylation of Akt will induce GLUT4 translocation (Izela et al. 2017). To further investigate the effects of surfactin on regulation of glucose metabolism, the mRNA expression of genes, and expression of protein associated with glucose metabolism were measured. The results showed that high insulin led to slight increase in mRNA expression of Akt (*p* > .05) (Figure 3a,b). After treating with surfactin, mRNA expression of PI3K and Akt in the cells was notably upregulated as compared with those in IR‐HepG2 cells (Figure 3a,b). AMPK, AMP‐dependent protein kinase, is necessary to maintain glucose homeostasis. As shown in Figure 3(c), surfactin treatment significantly increased mRNA expression of AMPK compared with that in IR‐HepG2 cells. However, western blot analysis indicated that protein expression of PI3K was significantly upregulated while the ratio of p‐PI3K to PI3K (p‐PI3K/PI3K) not significantly different after treating with surfactin, as compared with those in IR‐HepG2 cells (Figure 4b,e). The protein expression of AMPK and Akt was not significantly different (Figure 3d) or was downregulated (Figure 4c,d) in surfactin treatment group as compared with that in IR‐HepG2 cells. Western blot analysis also showed that ratios of p‐Akt to Akt (p‐Akt/Akt) and p‐AMPK to AMPK (p‐AMPK/AMPK) were significantly increased in surfactin treatment group as compared with that in IR‐HepG2 cells (Figure 4f,g). In addition, Li et al. reported that gymnemic acid had no effect on protein expression of AMPK but increased p‐AMPK/AMPK, further activating phosphorylation of AMPK (p‐AMPK), which also ameliorated hyperglycemia in T2DM. It was also reported that IR could be alleviated through upregulating p‐Akt and activating PI3K/Akt signaling pathway (Wang, He, et al., 2020). Thus, we assumed that surfactin may increase protein expression of p‐AMPK and p‐Akt, further ameliorating IR in HepG2 cells. The mRNA expression of upstream regulator of GLUT4, GLUT4, and regulator of gluconeogenesis (hepatocyte nuclear factor 4α, HNF4α) exhibited a similar trend as their corresponding proteins (Figure 3e–g). This indicated that surfactin played critical roles in ameliorating IR and inhibiting gluconeogenesis by suppressing expression of genes and proteins related to glucose metabolism. It is speculated that surfactin promoted GLUT4 translocation probably through activating the PI3K/Akt and AMPK signaling pathway, resulting in promotion of glucose consumption and amelioration of IR.

### 3.6 Effects of surfactin on ROS generation in IR‐HepG2 cells

**FIGURE 5:**
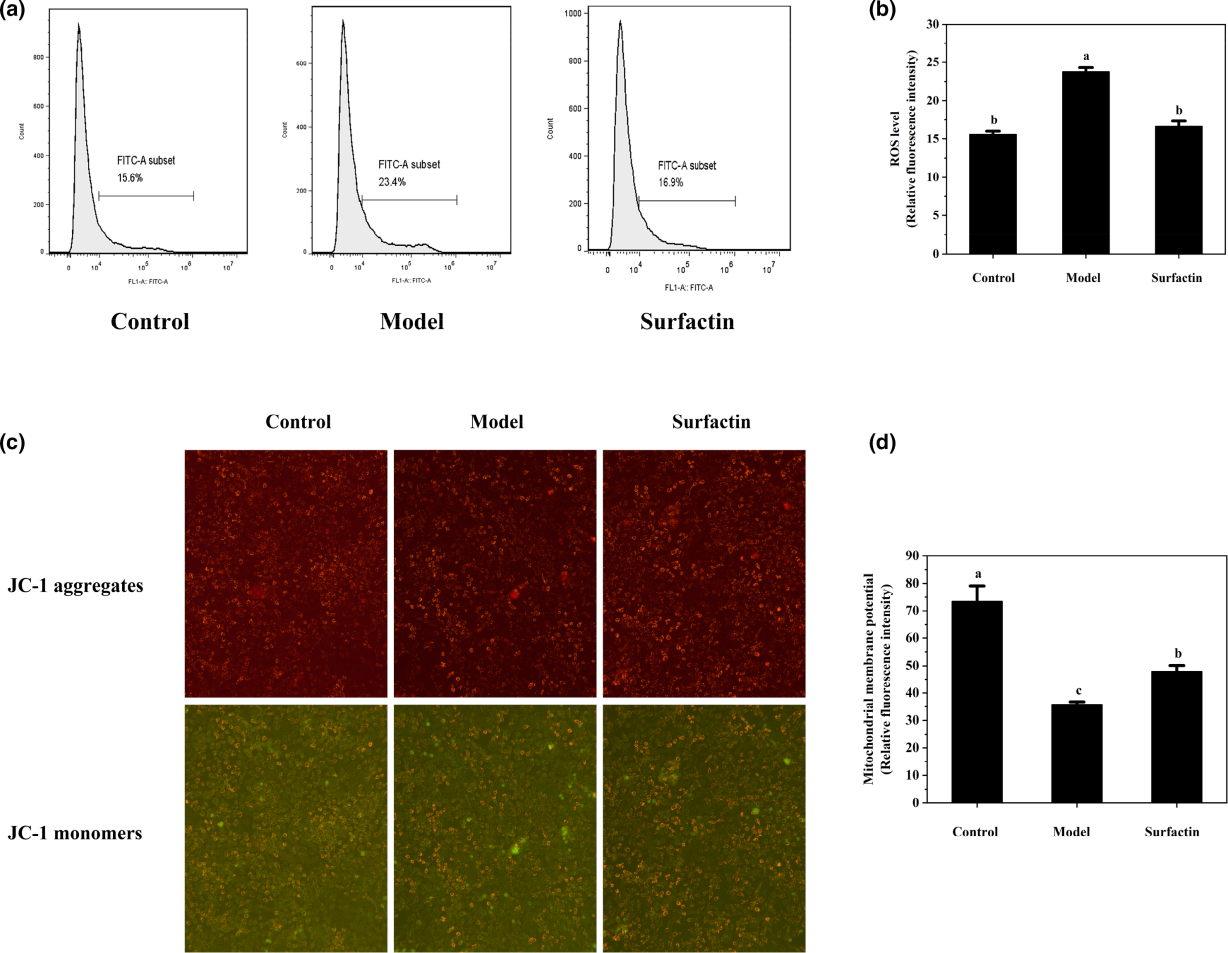
Effects of surfactin on ROS generation and mitochondrial membrane potential in IR‐HepG2 cells. (a) The mean fluorescence intensity reflects intracellular ROS level (10^4^−10^6^). (b) The corresponding histograms of DCFH‐DA fluorescence intensity. (c) Fluorescence images of JC‐1 aggregates and JC‐1 monomers. (d) Relative mitochondrial membrane potential after calculation of the ratio of JC‐1 aggregates to JC‐1 monomers. All data are presented as mean ± *SD* (*n* ≥ 3) for each group. Different lowercase alphabet letters over bars indicate statistically significant differences between two groups (*p* < .05)

Previous reports showed that oxidative stress is regarded as a major cause of IR development. High glucose induced excessive accumulation of ROS and eventually led to oxidative stress, which contributes to liver injury (Du et al., 2010). To investigate effects of surfactin on oxidative stress, the level of intracellular ROS was determined. As shown in Figure 5(a,b), high insulin dramatically increased ROS production by 52.2% (*p* < .05) as compared with that in control group. However, surfactin treatment decreased ROS production in the model group by 30.0% as compared with that in IR‐HepG2 cells (*p* < .05).

### 3.7 Effects of surfactin on mitochondrial membrane potential in IR‐HepG2 cells

As an important intracellular signal, mitochondrial membrane potential can initiate the antioxidative defense system to attenuate cellular oxidative stress (Chandel, 2015). As shown in Figure 5(c,d), the mitochondrial membrane potential in the model group was decreased by 51.3% as compared with that in the control group (*p* < .05), while surfactin treatment increased mitochondrial membrane potential by 33.7% as compared with that in the model group (*p* < .05).

### 3.8 Effects of surfactin on inflammatory responses in IR‐HepG2 cells

**FIGURE 6:**
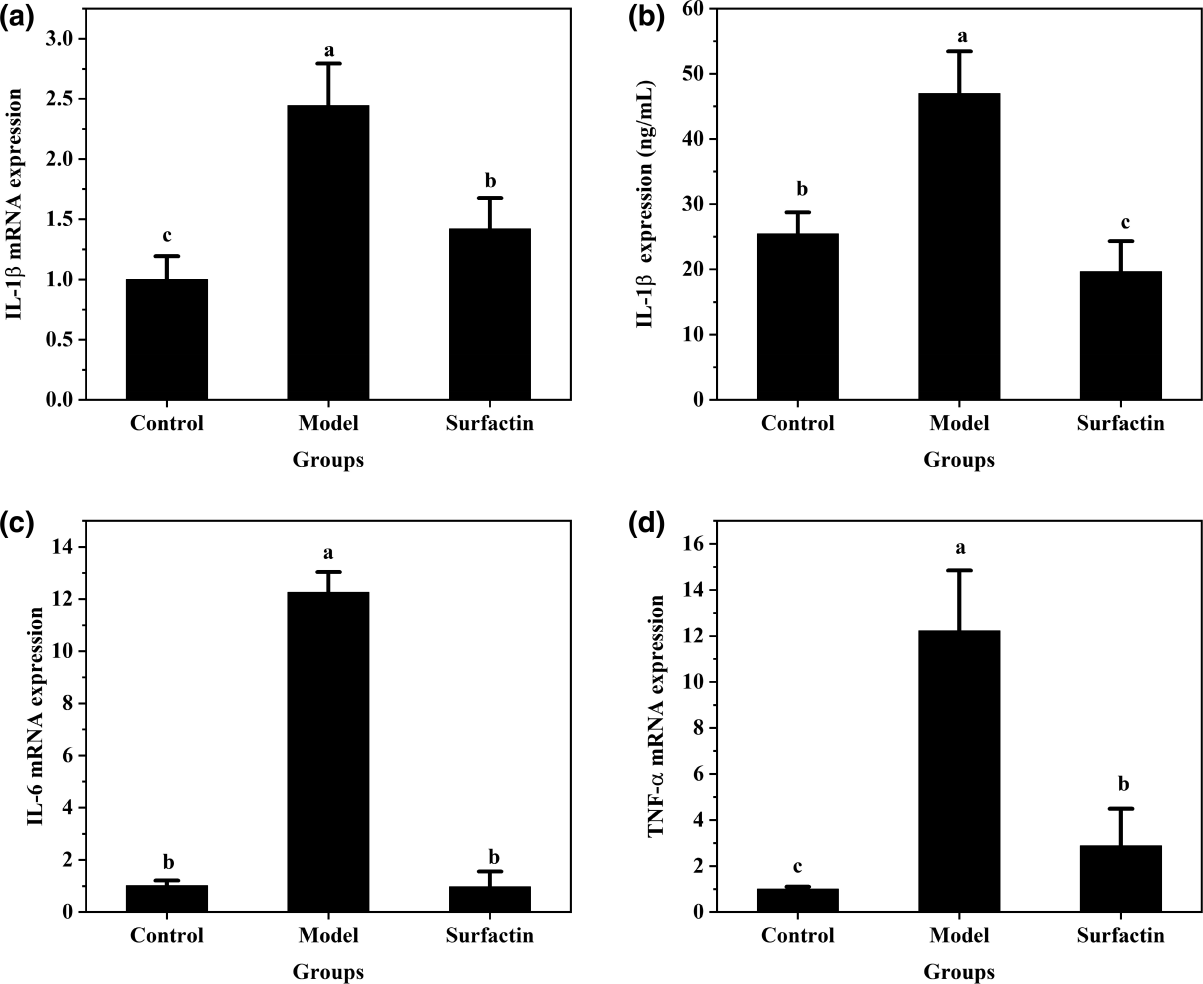
Effects of surfactin on mRNA and protein expression level of proinflammatory factors associated with insulin resistance signaling pathway in IR‐HepG2 cells. (a) The mRNA expression of IL‐1β. (b) The protein expression of IL‐1β. (c) The mRNA expression of IL‐6. (d) The mRNA expression of TNF‐α. All data are presented as mean ± *SD* (*n* = 6) for each group. Different lowercase alphabet letters over bars indicate statistically significant differences between two groups (*p* < .05)

The protein and gene expression of pro‐inflammatory factors were detected to evaluate effects of surfactin on inflammatory responses in IR‐HepG2 cells. As shown in Figure 6(a,b), high insulin significantly upregulated mRNA and protein expression of IL‐1β as compared with those in the control group. However, surfactin dramatically downregulated mRNA and protein expression of IL‐1β as compared with those in IR‐HepG2 cells (Figure 6a,b). In addition, insulin‐treated cells possessed a higher mRNA expression of IL‐6 and TNF‐α as compared with those in the control group (*p* < .05) (Figure 6c,d). Moreover, surfactin treatment significantly downregulated mRNA expression of IL‐6 and TNF‐α as compared with those of IR‐HepG2 cells (Figure 6c,d). These revealed that surfactin could inhibit proinflammatory cytokines release. These results are consistent with a previous report (Wang et al., 2021).

### 3.9 Effects of surfactin on the glucose consumption in Caco‐2 cells

**FIGURE 7:**
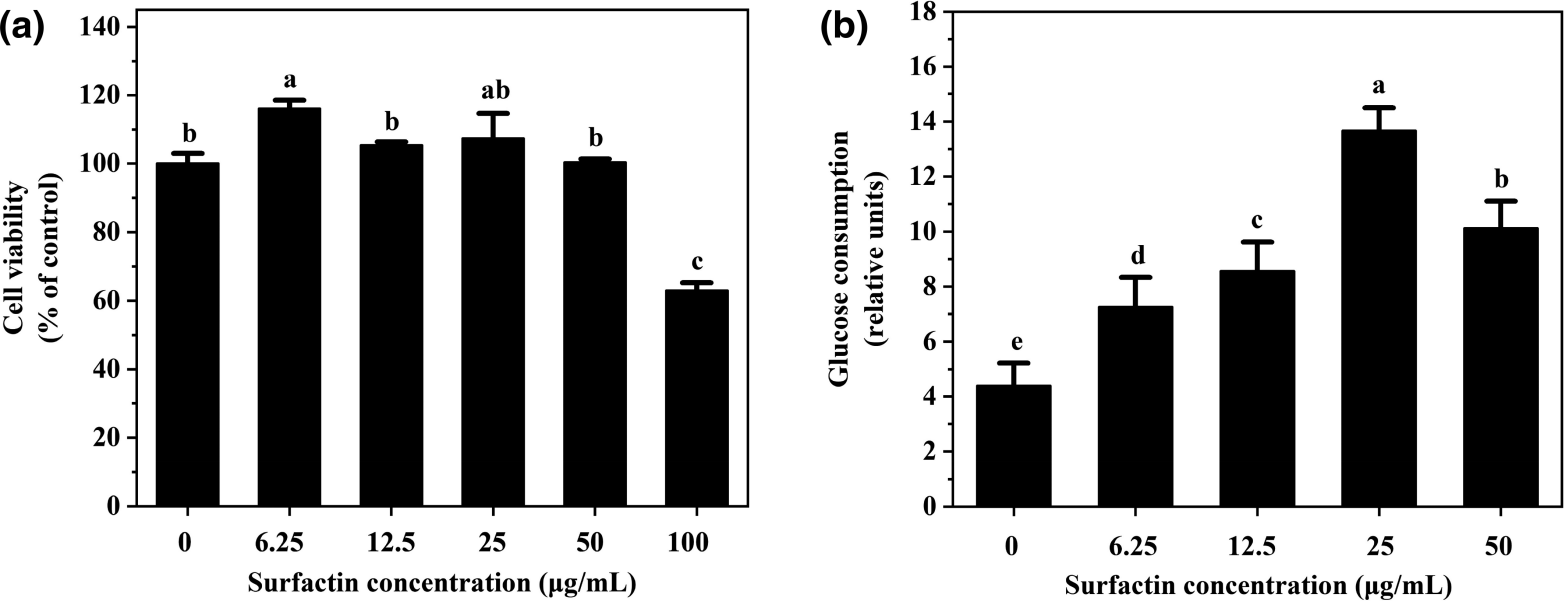
Effect of surfactin on cell viability and glucose consumption in Caco‐2 cells. (a) Cell viability with different concentrations (0–100 μg/ml) of surfactin for 48 h. (b) Glucose consumption without and with surfactin for 48 h. All data are expressed as mean ± *SD* (*n* = 6) for each group. Different lowercase alphabet letters were significantly different at level of *p* < .05

To explore the effects of surfactin on Caco‐2 cells, glucose consumption was also determined in Caco‐2 cells. As shown in Figure 7(a), 6.25–50 µg/ml of surfactin had no cytotoxic effect on Caco‐2 cells. Glucose consumption in Caco‐2 cells was obviously increased, when compared with that in the control group, in a dose‐wise pattern (Figure 7b). This revealed that surfactin also promoted glucose consumption in Caco‐2 cells. In addition, 12.5 µg/ml of surfactin as surfactin group was used for the following study in Caco‐2 cells.

### 3.10 Effects of surfactin on GLUT4 in Caco‐2 cells

**FIGURE 8:**
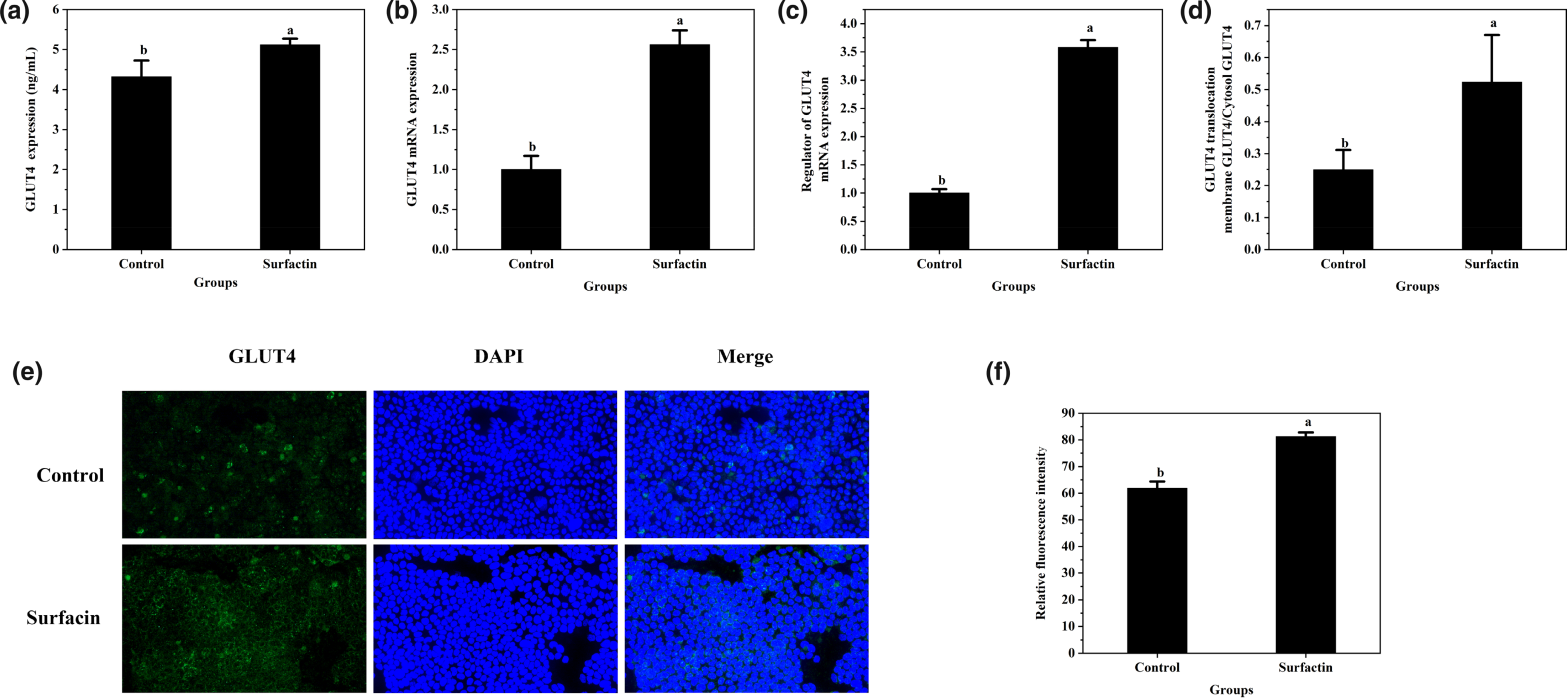
Effects of surfactin on GLUT4, its translocation, and mitochondrial membrane potential in Caco‐2 cells. (a) The protein expression of GLUT4. (b) The mRNA expression of GLUT4. (c) The mRNA expression of regulator of GLUT4. (d) The GLUT4 translocation. (e) Representative images of immunofluorescence staining of GLUT4. (f) The corresponding relative fluorescence intensity of GLUT4. All data are presented as mean ± *SD* (*n* ≥ 3) for each group. Different lowercase alphabet letters over bars indicate statistically significant differences between two groups (*p* < .05)

The protein expression and translocation of GLUT4 were detected in Caco‐2 cells. As shown in Figure 8(a), the protein expression of GLUT4 was significantly increased in surfactin treatment group compared with that in the control group (*p* < .05). The results of immunofluorescence staining also indicated that surfactin increased protein expression of GLUT4 in Caco‐2 cells (Figure 8e,f). The mRNA expression of GLUT4 and its regulator were notably upregulated after treating with surfactin as compared with those of the control group (Figure 8b,c). In addition, surfactin elevated the GLUT4 translocation by 109.9% as compared with that in the control group (*p* < .05) (Figure 8d). These indicated that surfactin also promoted protein expression and translocation of GLUT4 in Caco‐2 cells.

### 3.11 Effects of surfactin on body weight, food intake, and FBG in T2DM mice induced by STZ/HFD

**FIGURE 9:**
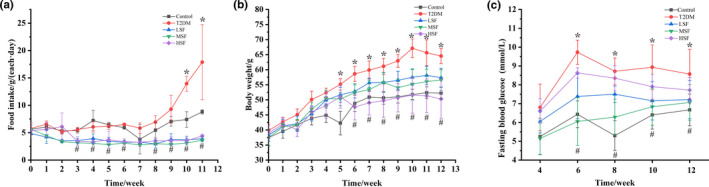
Effects of surfactin on body weight, food intake, and fasting blood glucose of T2DM mice induced by STZ/HFD. (a) Body weight. (b) Food intake. (c) Fasting blood glucose. All data are presented as mean ± *SD* (*n* = 10) for each group. *Indicates statistically significant differences between the control group and the T2DM group (*p* < .05). #Indicates statistically significant differences between the T2DM and surfactin supplement group (*p* < .05)

The body weight and food intake of mice were recorded during experiment. As shown in Figure 9(b), body weight of mice possessed an increase trend, and was stable after 8 weeks; while the body weight of the T2DM group significantly decreased after 9 weeks, as compared with that control and surfactin administration group (*p* < .05). The body weight in T2DM mice dramatically increased as compared with that in the control group (*p* < .05). Surfactin significantly decreased body weight of T2DM mice. As shown in Figure 9(a), food intake of mice increased in the control and T2DM group, while maintained stable in surfactin supplement group. In addition, food intake of T2DM mice was notably enhanced as compared with that of the control and surfactin group after 7 weeks. Furthermore, food intake in the surfactin groups dramatically lowered than that in the control group. These indicated that surfactin significantly inhibited food intake and maintained body weight stability.

The FBG of all mice was recorded every 2 weeks. As shown in Figure 9(c), the FBG of the T2DM group was dramatically increased compared with that of the control group (*p* < .05). After surfactin supplement, the FBG of mice in the MSF group was significantly decreased, when compared with that in the T2DM group (*p* < .05). The FBG of LSF and HSF group was not significantly different compared to that in T2DM group.

## 4 DISCUSSION

T2DM is a complex and chronic metabolic disease characterized by IR and hyperglycemia, and is also regarded as one of the main threats to human health (Li et al., 2019). In the basal state, Akt is phosphorylated after activating insulin‐mediated signaling pathway, and then plays roles in regulating glucose metabolism via multiple pathways. First, it promotes glycogen synthesis. Second, it promotes GLUT4 translocation and increases glucose uptake. Third, it inhibits gluconeogenesis via downregulating upstream regulator factor of gluconeogenic rate‐limiting enzyme (Chen et al., 2019). However, these functions might be attenuated in IR hepatic cells. The glucose transporters (GLUTs) are a group of intrinsic proteins in the plasma membrane, and helps the tissue glucose uptake under the influence of insulin (Yin et al., 2009). In particular, GLUT4 translocation from the cytoplasm to the membrane is the basis of glucose transport under the stimulation of insulin in hepatic cells. In addition, AMPK dysfunction caused by IR might also lead to inhibition of GLUT4 translocation. It is reported that the promotion of glucose uptake in skeletal muscle and liver involves two separate pathways: the insulin‐dependent mechanism such as PI3K/Akt activation and contraction‐mediated AMPK stimulation (Kuo et al., 2016). In this study, we observed that surfactin treatment increased hepatic glucose consumption. The following investigation indicated that surfactin upregulated mRNA expression of AMPK, increased p‐Akt/Akt and p‐AMPK/AMPK in the protein level, activated PI3K/Akt and AMPK pathway, and increased GLUT4 translocation, meanwhile suppressed protein expression of PEPCK and G6Pase. We speculated that surfactin promoted glucose consumption possibly owing to increasing GLUT4 translocation by activating PI3K/Akt and AMPK signaling pathways. Previous reports also indicated that phosphorylated AMPK and Akt could prevent hepatic gluconeogenesis and lower blood glucose levels, independent of the activation of IRS‐1/PI3K/Akt insulin signaling (Li et al., 2019; Wang et al., 2020b). This is consistent with our investigation. In addition, we observed that surfactin treatment did not affect hepatic glycogen synthesis (Figure S1a,b). Taken together, we concluded surfactin ameliorated IR by increasing glucose consumption, reducing gluconeogenesis, and activating the PI3K/Akt and AMPK signaling pathways (Figure 8). However, this hypothesis needs to be further verified.

Glucose transporters also play a major role in the effective absorption of glucose via the insulin‐mediated pathway in peripheral tissues, such as skeletal muscle and adipose tissue (Kuo et al., 2016). It is reported that increased glucose absorption of intestinal cells and decreased glucose into blood in the intestine–pancreatic axis also could alleviate IR (Yang et al., 2021). We also investigated the effects of surfactin on the glucose consumption in Caco‐2 cells. In fact, the peptides in human body might be broken into some small peptides by pepsin, trypsin, and chymotrypsin to weaken their bioactivities during the oral administration and gastrointestinal digestion (Wang et al., 2020a). In view of this, in vitro digestion of surfactin was performed, including oral administration and gastrointestinal digestion. The results indicated that surfactin could not be broken down and maintains initial bioactivity after digesting (Figures S2 and S3). In addition, it was observed that surfactin also increased the glucose consumption of Caco‐2 cells. Meanwhile, surfactin promoted GLUT4 translocation in Caco‐2 cells. However, we observed that surfactin treatment did not affect protein expression of AMPK in Caco‐2 cells (Figure S1c). Therefore, it is speculated that the effects of surfactin on promoting GLUT4 translocation in Caco‐2 cells were probably through phosphorylated AMPK or other novel ways. These results confirmed the effects of surfactin on IR‐HepG2 cells. Finally, we concluded that surfactin ameliorated IR in intestine–hepatic axis possibly by promoting glucose absorption of intestinal cells, inhibiting hepatic gluconeogenesis, and inducing glucose consumption of hepatic cells.

On the other hand, we observed that surfactin increased mRNA and protein expression of GLUT4 in HepG2, and the results of Caco‐2 cells also confirmed this conclusion. It is well known that glucose transport in insulin‐sensitive tissues is closely related to the protein expression of glucose transporters. It is reported that glucose uptake could be increased by directly regulating the expression of several genes involved in glucose metabolism such as GLUT1 and GLUT4 (Staels & Fruchart, 2005). Choi et al showed that Artepillin C increased the concentrations of GLUT4 in the cytosolic fraction by upregulating mRNA and protein expression of GLUT4, leading to increase in the concentration of GLUT4 on membrane without enhancing insulin signaling pathway. Thus, we speculated that surfactin promoted glucose consumption possibly via directly upregulating mRNA and protein expression of GLUT4 in hepatic and intestinal cells, independent of insulin and AMPK signaling pathways.

Mitochondria is essential for ATP production, and contributes to GLUT4 translocation and glucose uptake in cells (Osorio‐Fuentealba et al., 2013). However, oxidative stress caused by excessive ROS and free radicals leads to cell membrane damage and mitochondria dysfunction (Hervera et al., 2018), blocking insulin signaling through MAPK pathway. In this study, surfactin inhibited hepatic ROS overproduction, and increased mitochondrial membrane potential in HepG2, indicating that surfactin inhibited oxidative stress and restored mitochondria dysfunction. It is speculated that surfactin suppressed ROS overproduction and restored mitochondrial dysfunction probably through activating MAPK pathway, contributing to ameliorate IR. This speculation still needs further investigation to confirm. In addition, it is reported that protein hydrolysate of oysters could donate electrons to free radicals and convert them into more stable and less chemically reactive products (Wang et al., 2014). Peptides with high proportion of hydrophobic amino acids also exhibit high radical eliminating activities owing to their hydrophobic interaction with phospholipid bilayer of membrane (Wang et al., 2020a). Based on this, we hypothesized that surfactin eliminated ROS probably due to its high hydrophobic amino acid Leu.

In general, an imbalance in pro‐ and anti‐inflammatory factors aggravates metabolic inflammation and induces IR (Tang et al., 2021). Intracellular ROS accumulation might activate NF‐κB pathway (Tang et al., 2019) and release of TNF‐α and IL‐6, blocking IRS‐1/PI3K signaling and aggravating IR (Tang et al., 2019). In our study, it is observed that mRNA expression of IL‐6, TNF‐α, and IL‐1β, and protein expression of IL‐1β were suppressed after treating with surfactin in IR‐HepG2 cells. These results are in agreement with a previous study (Tang et al., 2020). These revealed that surfactin inhibited proinflammatory factors production and improved insulin signaling pathway.

At present, food‐derived bioactive ingredients are increasingly used to ameliorate IR owning to their nontoxic, non–side effects, and low cost, such as vinegar extracts (Xia et al., 2021), nut‐derived peptides (Wang et al., 2020b), and the postbiotics (Zouari et al., 2016). Common metformin and rosiglitazone could control hyperglycemia due to indirect activation of AMPK by disturbing mitochondrial respiration (Turner et al., 2008). It was reported that C‐phycocyanin, a kind of blue protein isolated from *Spirulina platensis*, can ameliorate hyperglycemia through inhibiting hepatic gluconeogenesis and increasing glycogen synthesis due to activating Akt and AMPK in insulin resistance hepatocytes (Ren et al., 2018). A postbiotic, the extracellular polysaccharide extracted from *Lactobacillus plantarum* RJF4 can inhibit the activity of α‐glucosidase, further lowering blood glucose and playing important roles in antidiabetes (Dilna et al., 2015). A previous report showed that surfactin produced from *B. amyloliquefaciens* WH1 is regarded as a novel drug for alleviating T1DM (Gao et al., 2014). Surfactin also decreased serum total cholesterol (TC) and low‐density lipoprotein cholesterol (LDL‐C), and increased serum high‐density lipoprotein cholesterol (HDL‐C) in mice. These reports revealed that surfactin possessed antidiabetic effects. However, surfactin exhibited beneficial effects on regulation of glucose consumption, the protein expression, and translocation of GLUT4 and gluconeogenesis, in addition to elimination of ROS and inhibition of proinflammatory factors in this study. On the other hand, surfactin also could improve oral delivery of insulin via increasing its penetration through the cell membrane (Zhang et al., 2016). This indicated that surfactin as an insulin carrier can maintain insulin stability and facilitate insulin delivery to target sites. However, this displays a different action compared to this study. In our study, surfactin as a postbiotic promoted hepatic glucose consumption through regulating PI3K/Akt/GLUT4 and AMPK signaling pathways, further regulating glucose metabolism and ameliorating IR. In addition, it was observed that surfactin supplement also significantly lowered fasting blood glucose of T2DM mice induced by STZ/HFD. However, the detail mechanism still needs to be investigated in animal model.

## 5 CONCLUSION

**FIGURE 10:**
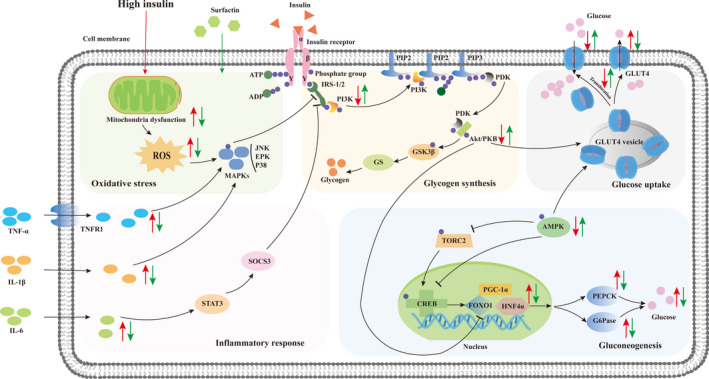
Underlying and hypothesis of molecular mechanism ameliorating IR of surfactin in IR‐HepG2 cells. Red arrows denote changes in response to high insulin, and green arrows denote changes in high insulin‐induced cells receiving surfactin intervention

In conclusion, surfactin could ameliorate IR probably owing to promotion of GLUT4 translocation and suppression of protein expression of PEPCK and G6Pase through activation of PI3K/Akt and AMPK signaling pathways, reduction in pro‐inflammatory factors, and oxidative stress in hepatic cells (Figure 10) and activation of GLUT4 translocation in Caco‐2 cells. On the other hand, surfactin promoted glucose consumption possibly through directly upregulating protein expression of GLUT4 in hepatic and Caco‐2 cells, independent of PI3K/Akt and AMPK pathways. Furthermore, surfactin also lowered body weight, food intake, and FBG of T2DM mice induced by STZ/HFD. Therefore, surfactin can attenuate high insulin‐induced IR and decrease high blood glucose. It is encouraging for further exploration into amelioration of IR and regulation effects on glucose metabolism in animals and humans.

## CONFLICT OF INTEREST

The authors declare no competing financial interest.

## AUTHOR CONTRIBUTION

**Xiaoyu Chen:** Data curation (equal); Formal analysis (equal); Writing – original draft (equal). **Hongyuan Zhao:** Data curation (equal). **Fanqiang Meng:** Writing – review & editing (equal). **Libang Zhou:** Writing – review & editing (equal). **xinyi Pang:** Writing – review & editing (equal). **Zhaoxin Lu:** Project administration (equal). **Yingjian Lu:** Writing – review & editing (equal).

## Supporting information

Supplementary Material
