# Supplementary material for: Ameliorated effects of a lipopeptide surfactin on insulin resistance in vitro and in vivo
Source: Food Sci Nutr. 2022 Mar 29;10(7):2455–69. doi: 10.1002/fsn3.2852 (PMC9281957; doi:10.1002/fsn3.2852)

**Simulated saliva digestion**

The simulated saliva digestion was performed according to the reported method (Zhou et al. 2018) with slight modification. The fresh human saliva was collected from a healthy volunteer who was without antibiotics treatment in the last three months. Attentively, the volunteer rinsed their mouth and discarded the initial 30 s saliva before collection. The collected saliva was centrifuged (4000 rpm, 10 min) immediately, and the supernatant was used for simulated saliva digestion. The amylase activity of saliva was measured by the reported method of van Ruth and Roozen (28 U/mL) (Ruth et al. 2000). 2.5 mg/mL of surfactin solution were used. Subsequent step as following in Table S1.

**Table S1**. The steps of saliva digestion *in vitro*.

| List | Tube A | Tube B | Tube C |
| --- | --- | --- | --- |
| Surfactin/mL | 4 |  | 4 |
| Saliva/mL | 4 | 4 |  |
| Deionized water/mL |  | 4 | 4 |
| All tubes were incubated in a water bath oscillator of 37℃,120 rpm. | | | |

During the digestion (at 0, 1, 2, 4h), 2.0 mL mixture was taken out to further analysis and immersed immediately into a boiling water bath for 5 min to inactivate amylase. Each experiment was repeated three times.

**Simulated Gastric Digestion**

The simulated gastric digestion was explored as following the reported method (Zhou et al. 2018) with some modifications. Briefly, 620.0 mg NaCl, 225.0 mg KCl, 30.0 mg CaCl2 and 120.0 mg NaHCO_3_ was prepared as gastric electrolyte solution (GES, 200 mL), and the pH was adjusted to 3.0 using HCl solution (0.1M). The simulated gastric juice is composed of 23.6 mg pepsin, 25.0 mg gastric lipase, 1.0 mL CH3COONa solution (1.0 M, pH 5.0) and 100.0 mL GES, and the pH is adjusted to 3.0 using HCl solution (0.1 M). 25 mg/mL of surfactin solution were used. Subsequent step as following in Table S2.

**Table S2**. The steps of gastric digestion *in vitro*.

| List | Tube A | Tube B | Tube C |
| --- | --- | --- | --- |
| Surfactin/mL | 8 |  | 8 |
| Gastric juice/mL | 8 | 8 |  |
| Deionized water/mL |  | 8 | 8 |
| All tubes were incubated in a water bath oscillator of 37℃,120 rpm. | | | |

During the digestion (at 0, 2, 4, 6h), 3.0 mL mixture was taken out to further analysis and immersed immediately into a boiling water bath for 5 min to inactivate amylase. Each experiment was performed in triplicate.

**Simulated Small Intestinal Digestion**

The simulated small intestinal juice was assembled according to the method described in previous report with little modification (Chen et al. 2018). 1.08 g of NaCl, 130.0 mg of KCl, and 48.0 mg of CaCl2 was prepared as intestinal electrolyte solution (IES, 200 mL), and the pH was adjusted to 7 by NaOH solution (0.1 M). The simulated small intestinal juice is consisting of 100 g of pancreatin solution (7%, w/w), 6.5 mg of trypsin, 200 g bile salt solution (4%, w/w) and 100 mL of IES. And the pH was adjusted to 7.5 by NaOH solution (0.1 M). Subsequently, the pH of gastric digested solution at 6 h of digestion was adjusted to 7.0. The simulated small intestinal digestion as following in Table S3.

**Table S3**. The steps of small intestinal digestion *in vitro*.

| List | Tube A | Tube B | Tube C |
| --- | --- | --- | --- |
| Digested gastric juice /mL | 10 | 10 |  |
| Small intestinal juice/mL | 3 |  | 3 |
| Deionized water/mL |  | 3 | 10 |
| All tubes were incubated in a water bath oscillator of 37℃,120 rpm. | | | |

During the digestion, 1.0 mL of digested sample was taken out at 0, 2, 4, 6 h, respectively, an immersed immediately into a boiling water bath for 5 min to inactivate the enzymes. Each experiment was repeated three times.

**The biological activity of digested surfactin detection**

The indicator bacteria of *Staphylococcus aureus* ATCC 25923 and *Escherichia coli* ACTT 25922 were incubated in a shaker of 37℃,180 rpm. The absorbance at 600 nm of culture broth of two indicator bacteria were up to 0.4, which was used to further antibacterial activity detection. Subsequently, the mixtures of 100 μL of culture broth of the indicator bacteria with 100 μL of digested saliva, gastric juice and small intestinal juice were prepared at 96 well plate, respectively. The mixture of 100 μL deionized water with 100 μL of digested saliva, 100 μL of digested gastric juice and 100 μL of digested small intestinal juice were prepared respectively as control. All plates were incubated 37℃ for 12h and the absorbance at 600 nm was detected by a microplate reader.

**References**

Chen G. J., Xie M.H., Wan P., Chen D., Ye H., Chen L. G., Zeng X. X. and Liu Z. H. (2018) Digestion under saliva, simulated gastric and small intestinal conditions and fermentation in vitro by human intestinal microbiota of polysaccharides from Fuzhuan brick tea. *Food Chemistry* 244, 331-339.

Ruth S.M., Roozen J. P. Influence of mastication and saliva on aroma release in a model mouth system. *Food Chemistry*. 2000, 71, 339-345.doi:10.1016/S0308-8146(00)00186-2.

Zhou W.T., Yan Y. M., Mi J., Zhang H. C., Lu L., Luo Q., Li X. Y., Zeng X. X., Cao Y. L. Simulated Digestion and Fermentation in Vitro by Human Gut Microbiota of Polysaccharides from Bee Collected Pollen of Chinese Wolfberry. *Journal of Agricultural and Food Chemistry*. 2018, 66, 898-907. doi:10.1021/acs.jafc.7b05546.

FIGURE CAPTIONS

Figure S1. Effects of surfactin on glycogen and GK in IR-HepG2 cells. (A) The concentration of glycogen. (B) The protein expression level of GK. All data are expressed as mean ± SD (n=3) for each group. Different lowercase alphabet letters were significantly different at level of p < 0.05.

The resulted showed that glycogen and the protein expression level of GK in surfactin treatment group was not significantly difference compared with that high insulin-induced HepG2 cells.

Figure S2. High performance liquid chromatograms (HPLC) of surfactin after digesting in saliva, simulated gastric juice and simulated small intestinal juice. (A) The HPLC of surfactin in saliva. A1-3 represent HPLC of saliva, surfactin and digested surfactin in saliva for 0, 1, 2 and 4 h, respectively. (B) The HPLC of surfactin in simulated gastric juice. B1-3 represent HPLC of gastric juice, surfactin and digested surfactin in gastric juice for 0, 2, 4 and 6 h, respectively. (C) The HPLC of surfactin in simulated small intestinal juice. C1-3 represent HPLC of small intestinal juice, surfactin and digested surfactin in small intestinal juice for 0, 2, 4 and 6 h, respectively.

The results indicated that surfactin could not be digested by saliva, simulated gastric juice and simulated small intestinal juice.

Figure S3. The biological activity of surfactin after digesting in saliva, simulated gastric juice and simulated small intestinal juice. (A) The antibacterial activity of surfactin-digested in saliva. A1, A2 represent *Staphylococcus aureus* ATCC 25923 and *Escherichia coli* ACTT 25922 as indicator bacteria, respectively. (B) The antibacterial activity of surfactin-digested in simulated gastric juice. B1, B2 represent *Staphylococcus aureus* ATCC 25923 and *Escherichia coli* ACTT 25922 as indicator bacteria, respectively. (C) The antibacterial activity of surfactin-digested in simulated small intestinal juice. C1, C2 represent *Staphylococcus aureus* ATCC 25923 and *Escherichia coli* ACTT 25922 as indicator bacteria, respectively. Different lowercase alphabet letters were significantly different at level of p < 0.05.

The results illustrated that surfactin possessed a powerful antibacterial (*Staphylococcus aureus* ATCC 25923 and *Escherichia coli* ACTT 25922) activity after digesting in saliva, simulated gastric juice and simulated small intestinal juice.

Figure S4. A control for the immunofluorescence GLUT4. (a) GLUT4 for HepG2 cells. (b) GLUT4 for Caco-2 cells.

Figure S5. The hemolytic activity of surfactin. Columbia Agar Base were used to detect hemolytic activity of surfactin.

Figure S1


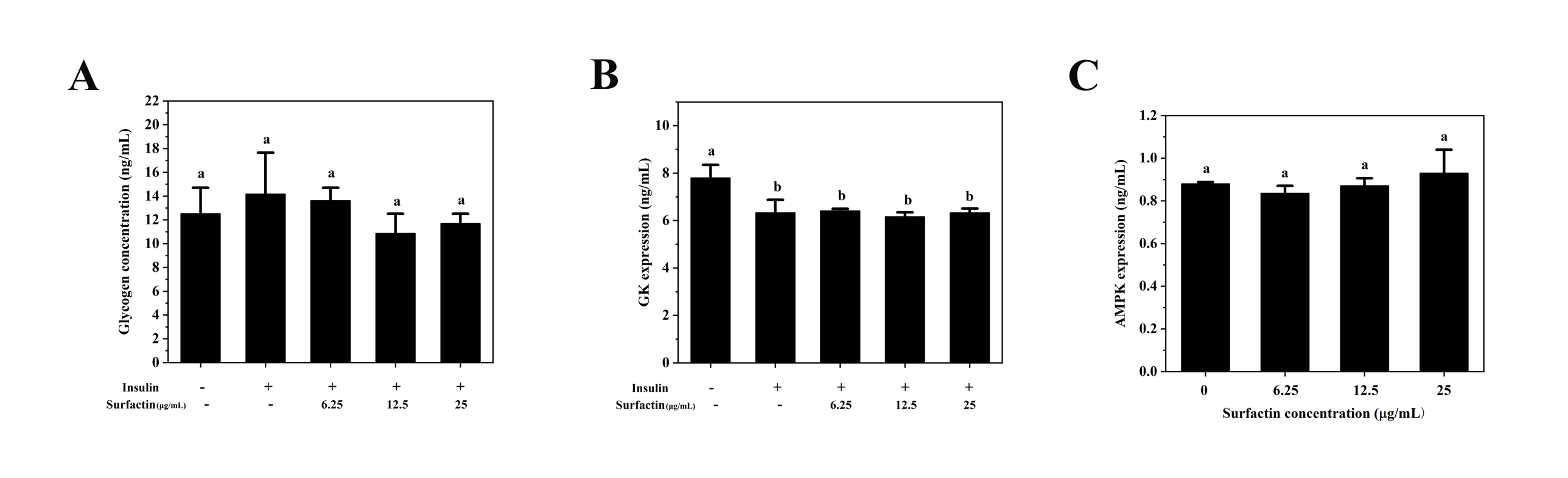


Figure S2


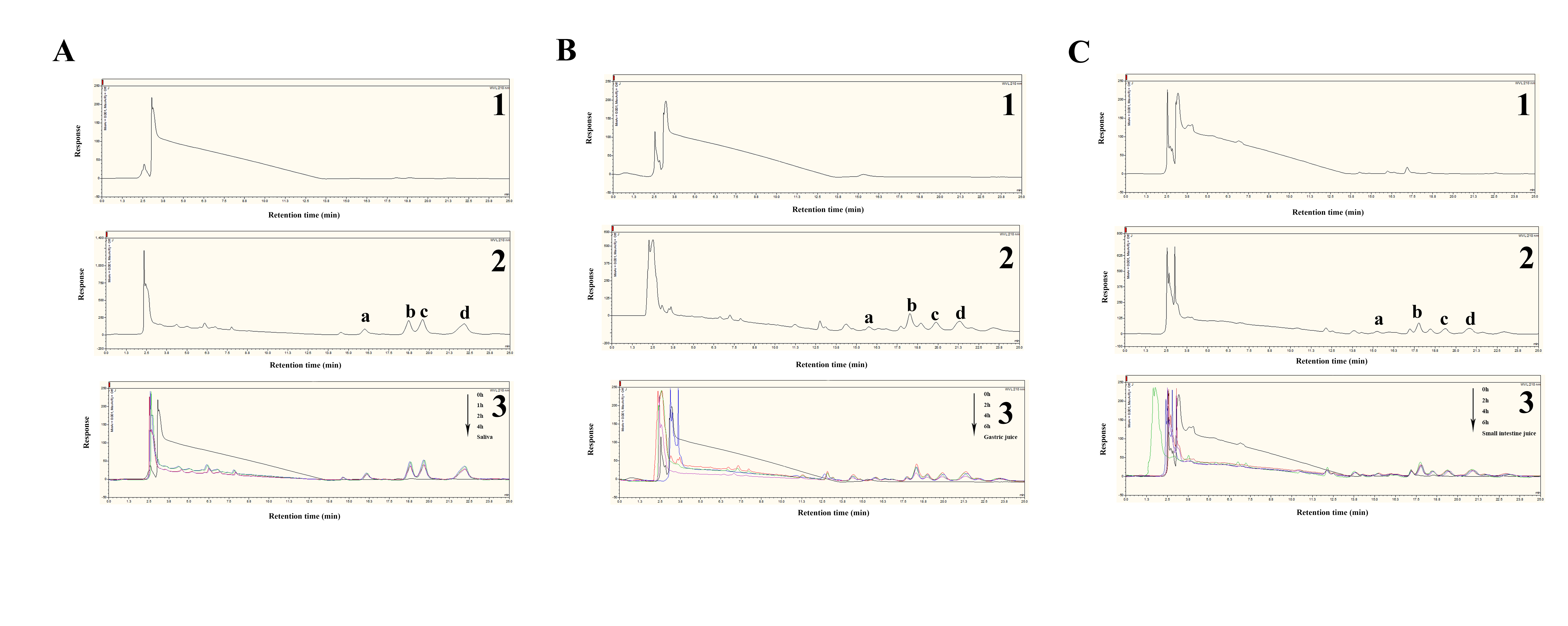


Figure S3


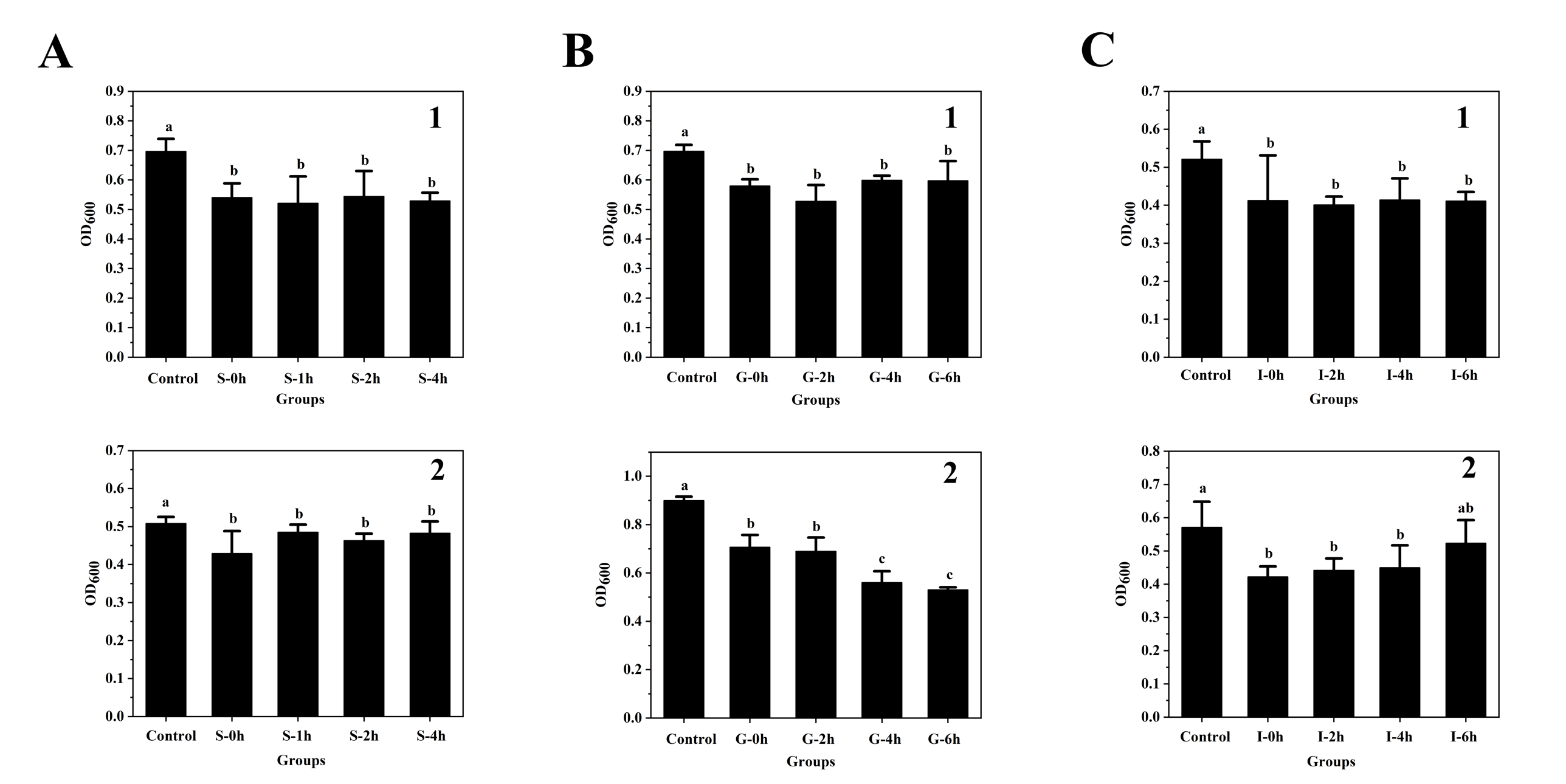


Figure S4


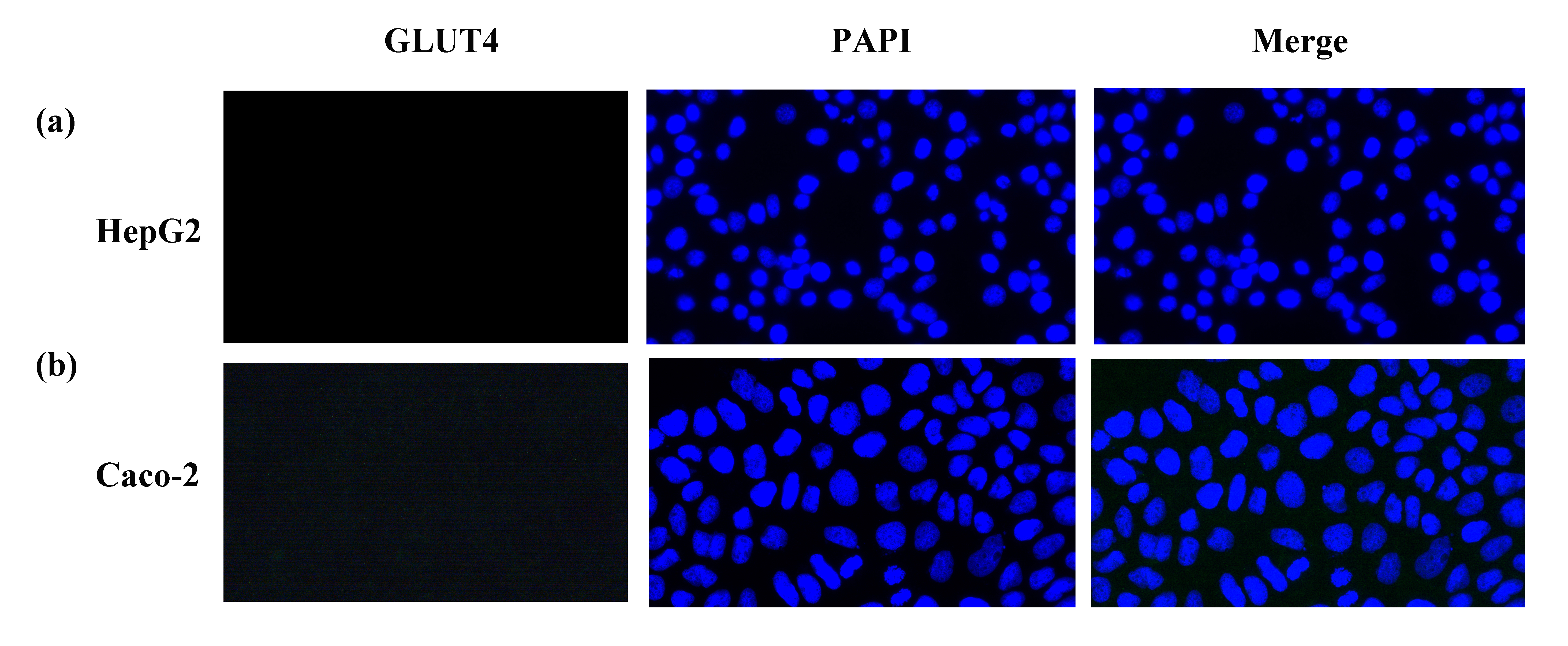


Figure S5


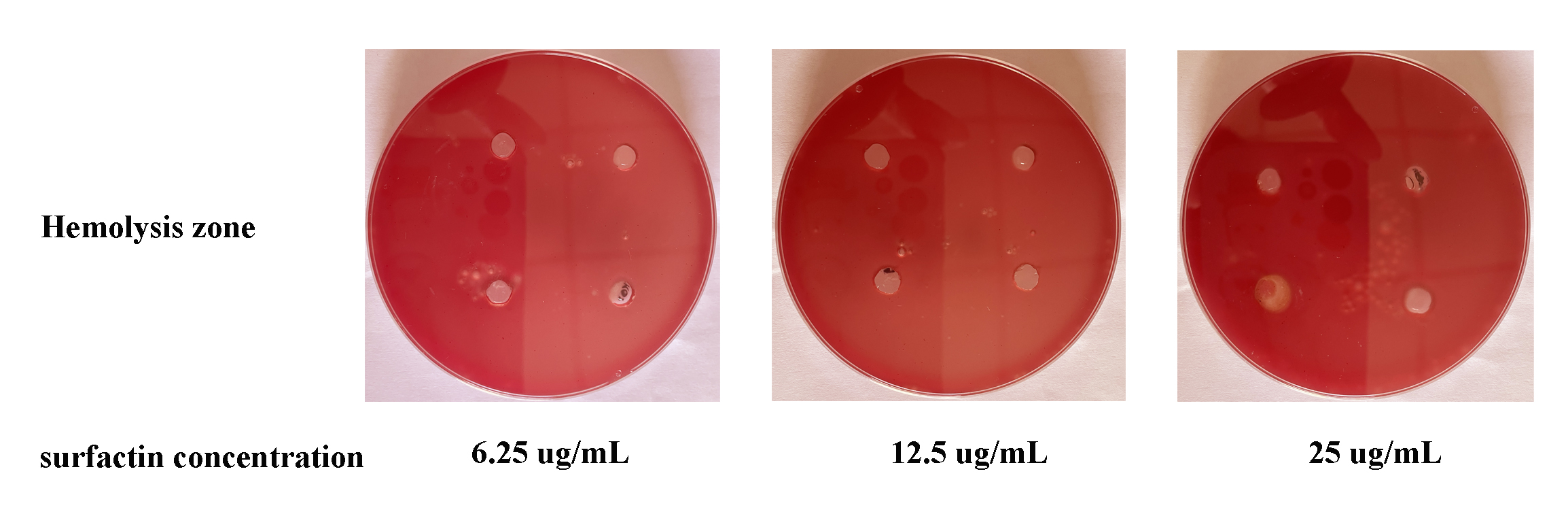

Supplement: Supplementary file 1 — Supplementary Material [file FSN3-10-2455-s001.docx]
